# Can a Woman Remain Ignorant of Her Pregnancy up to the Time of Her Delivery?

**Published:** 1860-09

**Authors:** James Duncan

**Affiliations:** Henrietta-sreet, Covent-Garden


### CAN A WOMAN REMAIN IGNORANT OF HER PREGNANCY UP TO THE TIME OF HER DELIVERY?

To the Editor of the London Lancet.

Sir,—The following case I can vouch for as decisively affirmative upon the above medico-legal point;—

On the 24th ultimo I was summoned to Mrs. R——, aged twenty-eight, married, with one child four years old. She stated that for the last twelve-months she had been ailing, and considered to be in a consumption; that a month or two before last Christmas she began to vomit blood, and, continuing to do so, she in March last consulted Dr. Tyler Smith, who, to her great surprise, pronounced her to be seven months pregnant. Up to the time of her labor the catamenia had been quite regular; she had suffered from none of the ordinary sympathetic ailments; had been sensible of no alterations in the breasts; and, notwithstanding Dr. Tyler Smith's opinion, she had not been able to detect any enlargement of her abdomen, or any sensation whatever of the child's movements. Had she not been informed of her pregnancy, she would undoubtedly have remained ignorant of her situation to the very last.

The liquor amnii was abundant, and the child a full-grown lively boy.

I am, Sir, yours, &c.,

James Duncan, M. D.

Henrietta-sreet, Covent-Garden,
July 12th, 1860.

We have known similar cases in which married women, who had previously borne children, did not believe they were pregnant until delivery was at hand.—Ed. L.

